# Supplementary material for: Formin tails act as a switch, inhibiting or enhancing processive actin elongation
Source: J Biol Chem. 2023 Dec 12;300(1):105557. doi: 10.1016/j.jbc.2023.105557 (PMC10797183; doi:10.1016/j.jbc.2023.105557)
Supplement: Supporting information [file mmc1.docx]

**Supporting Information for**

**Formin tails act as a switch, inhibiting or enhancing processive actin elongation**

Kathryn V. Bremer, Carolyn Wu, Aanand . Patel, Kevin L. He, Alex M. Grunfeld, Guillaume F. Chanfreau, Margot E. Quinlan

Material included:

Figure S1. Oxford Nanopore sequencing – samples of reads mapped to the *fhos* gene for the five developmental stages assessed.

Figure S2. Fhod variants that accumulated, which have not been previously annotated.

Tables 1-4: Summary of statistical analysis.

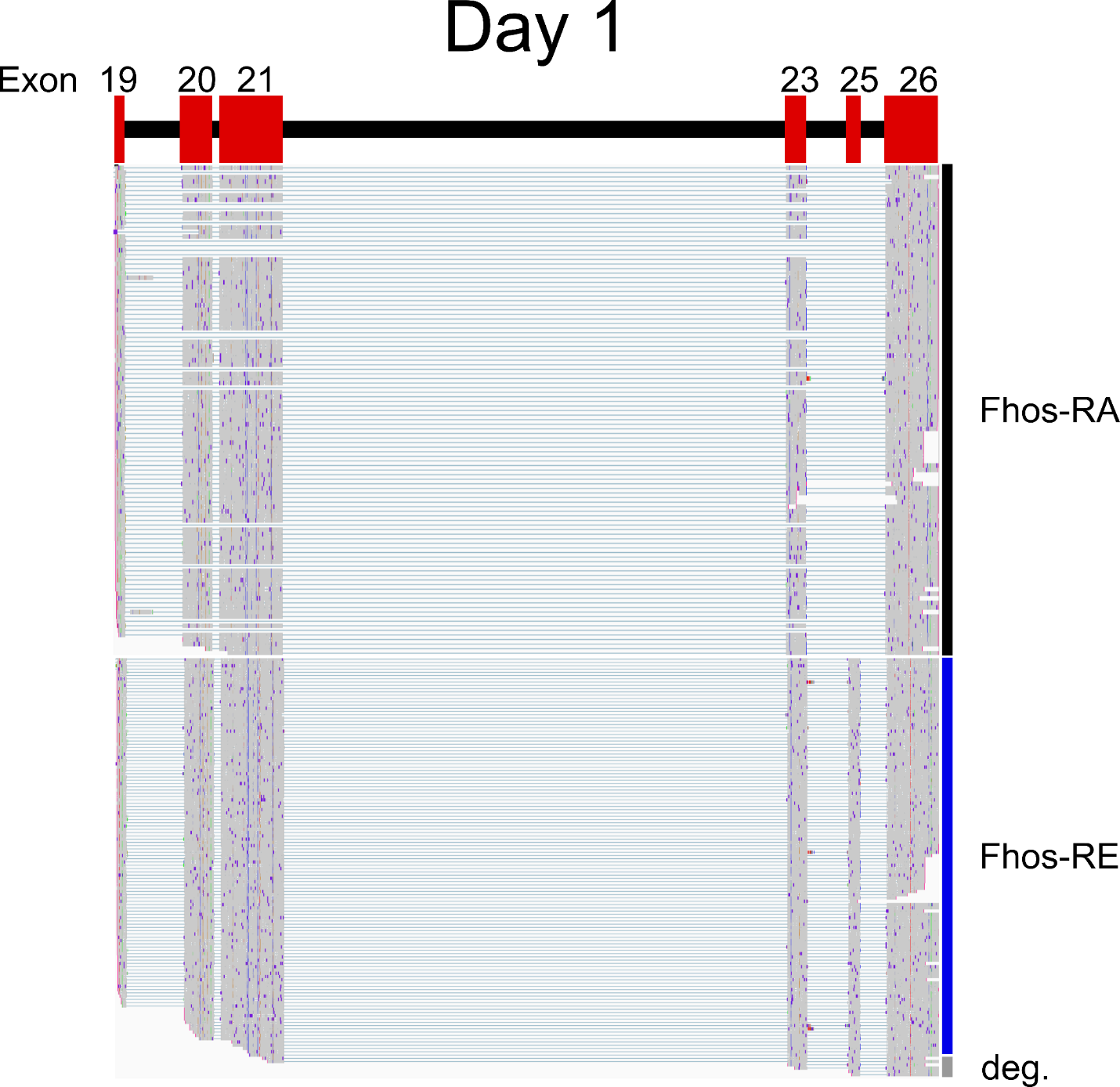


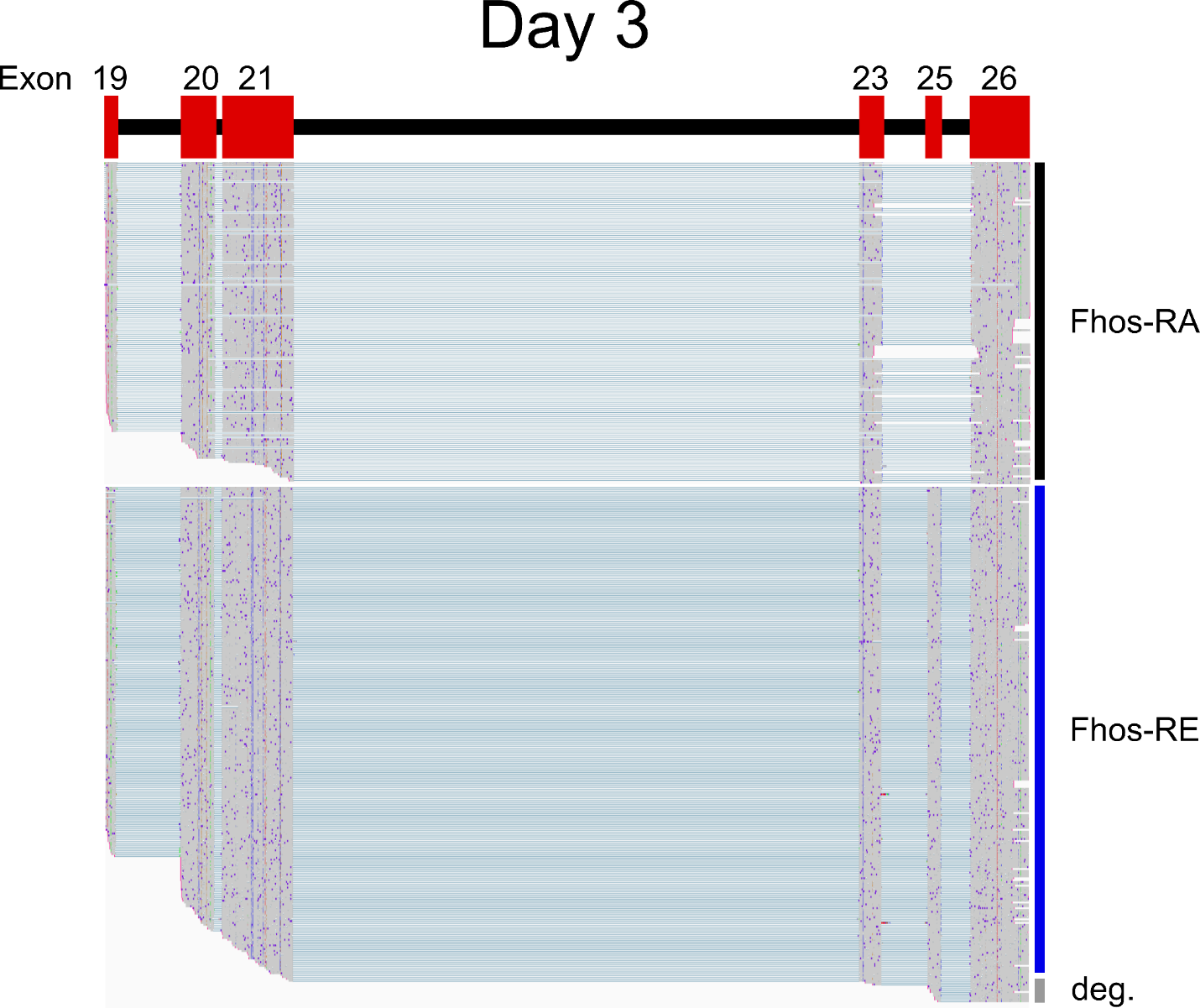

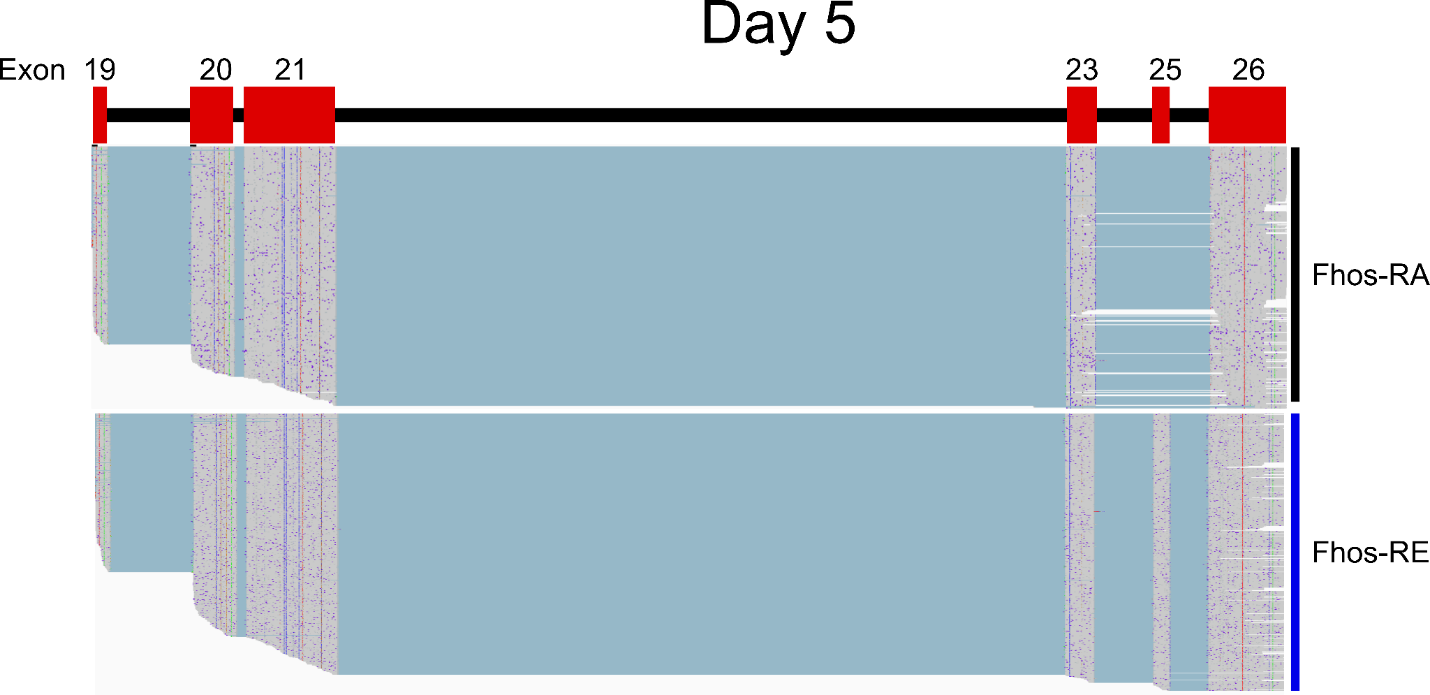


Figure S1. Oxford Nanopore sequencing – samples of reads mapped to the *fhos* gene for the five developmental stages assessed.

Figure S2. Fhod variants that accumulated, which have not been previously annotated. A) Detection of reads that show the extended region of exon 19. B) Detection of reads that terminate after exon 21.

Statistical analysis for Figure 2:

Table 1. Bright vs. dim filaments

| *Groups* | *Average*  *(bright)* | *Stdev*  *(bright)* | *Count*  *(bright)* | *Average*  *(dim)* | *Stdev*  *(dim)* | *Count (dim)* | *P-value^a^* |
| --- | --- | --- | --- | --- | --- | --- | --- |
| Actin alone | 5.46 | 0.96 | 16 | - | - | 0 | na |
| Fhod-A | 7.44 | 0.89 | 9 | - | - | 0 | na |
| Fhod-B | 3.77 | 1.47 | 6 | 16.70 | 2.51 | 10 | 1.81 x10^-8^ |
| Fhod-E | 4.85 | 1.92 | 19 | - | - | 0 | na |

^a^Students t-test, two tails, two sample equal variance

Tables 2A-C present results of one-way ANOVA analysis with Tukey Kramer Post Hoc test.

Table 2A. Summary

| *Groups* | *Count* | *Sum* | | *Average* | *Variance* |  |  |
| --- | --- | --- | --- | --- | --- | --- | --- |
| Actin alone | 16 | 87.30 | | 5.46 | 0.92 |  |  |
| Fhod-A | 9 | 67.00 | | 7.44 | 0.79 |  |  |
| Fhod-B | 10 | 167.00 | | 16.70 | 6.32 |  |  |
| Fhod-E | 19 | 92.10 | | 4.85 | 3.69 |  |  |
|  |  |  | |  |  |  |  |
| Table 2B. ANOVA |  |  | |  |  |  |  |
| *Source of Variation* | *SS* | | *df* | *MS* | *F* | *P-value* | *F crit* |
| Between Groups | 1045.64 | 3.00 | | 348.55 | 121.47 | 0.00 | 2.79 |
| Within Groups | 143.47 | 50.00 | | 2.87 |  |  |  |
|  |  |  | |  |  |  |  |
| Total | 1189.11 | 53.00 | |  |  |  |  |
|  |  |  | |  |  |  |  |

Table 2C. Tukey Kramer Post Hoc Test

| *Group 1* | *Group 2* | *diff* | *SE* | *Q* | *p < 0.05*  *Qcrit (4,50)=3.76* | *p < 0.01*  *Qcrit (4,50)=4.63* |
| --- | --- | --- | --- | --- | --- | --- |
| Actin alone | Fhod-A | 1.99 | 0.50 | 3.98 | y | n |
|  | Fhod-B | 11.24 | 0.48 | 23.29 | y | y |
|  | Fhod-E | 0.61 | 0.41 | 1.50 | n | n |
| Fhod-A | Fhod-B | 9.26 | 0.55 | 16.82 | y | y |
|  | Fhod-E | 2.60 | 0.48 | 5.36 | y | y |
| Fhod-B | Fhod-E | 11.85 | 0.47 | 25.33 | y | y |

Statistical analysis for Figure 3.

Table 3. Bright vs. dim filaments

| *Groups* | *Average (bright)* | *Stdev (bright)* | *Count (bright)* | *Average (dim)* | *Stdev (dim)* | *Count (dim)* | *P-value^a^* |
| --- | --- | --- | --- | --- | --- | --- | --- |
| Actin Alone | 5.46 | 0.96 | 16 | - | - | 0 | na |
| Fhod-A | 7.44 | 0.89 | 9 | - | - | 0 | na |
| Fhod-AΔ15 | 4.88 | 0.85 | 11 | - | - | 0 | na |
| Fhod-AΔ24 | 4.40 | 2.26 | 6 | 13.28 | 2.31 | 12 | 8.38 x 10^-7^ |
| Fhod-AΔ50 | 8.87 | 0.40 | 3 | 14.62 | 2.38 | 12 | 0.13 x 10^-3^ |
| Fhod-AΔ75 | 4.62 | 1.03 | 4 | 14.13 | 2.76 | 16 | 3.07 x 10^-6^ |
| Fhod-B | 3.77 | 1.47 | 6 | 16.70 | 2.51 | 10 | 1.82 x 10^-8^ |

^a^Students t-test, two tails, two sample equal variance

Tables 4A-C present results of one-way ANOVA analysis with Tukey Kramer Post Hoc test.

Table 4A. Summary

| *Groups* | *Count* | *Sum* | *Average* | *Variance* |  |  |
| --- | --- | --- | --- | --- | --- | --- |
| Actin Alone | 16 | 87.30 | 5.46 | 0.92 |  |  |
| Fhod-A | 9 | 67.00 | 7.44 | 0.79 |  |  |
| Fhod-AΔ15 | 11 | 53.69 | 4.88 | 0.71 |  |  |
| Fhod-AΔ24 | 12 | 159.40 | 13.28 | 5.33 |  |  |
| Fhod-AΔ50 | 12 | 175.50 | 14.63 | 5.67 |  |  |
| Fhod-AΔ75 | 16 | 226.10 | 14.13 | 7.64 |  |  |
| Fhod-B | 10 | 167.00 | 16.70 | 6.32 |  |  |
|  |  |  |  |  |  |  |
|  |  |  |  |  |  |  |
| Table 4B. ANOVA |  |  |  |  |  |  |
| *Source of Variation* | *SS* | *df* | *MS* | *F* | *P-value* | *F crit* |
| Between Groups | 1718.25 | 6 | 286.37 | 70.76 | 0.00 | 2.22 |
| Within Groups | 319.74 | 79 | 4.05 |  |  |  |
|  |  |  |  |  |  |  |
| Total | 2037.99 | 85 |  |  |  |  |

Table 4C. Tukey Kramer Post Hoc Test

| *Group 1* | *Group 2* | *diff* | *SE* | *Q* | *p < 0.05*  *Qcrit (7,79)=4.28* | *p < 0.01*  *Qcrit (7,79)=5.07* |
| --- | --- | --- | --- | --- | --- | --- |
| Actin Alone | Fhod-A | 1.99 | 0.59 | 3.35 | n | n |
|  | Fhod-AΔ15 | 0.58 | 0.56 | 1.03 | n | n |
|  | Fhod-AΔ24 | 7.83 | 0.54 | 14.41 | y | y |
|  | Fhod-AΔ50 | 9.17 | 0.54 | 16.88 | y | y |
|  | Fhod-AΔ75 | 8.68 | 0.50 | 17.25 | y | y |
|  | Fhod-B | 11.24 | 0.57 | 19.61 | y | y |
| Fhod-A | Fhod-AΔ15 | 2.56 | 0.64 | 4.01 | n | n |
|  | Fhod-AΔ24 | 5.84 | 0.63 | 9.31 | y | y |
|  | Fhod-AΔ50 | 7.18 | 0.63 | 11.45 | y | y |
|  | Fhod-AΔ75 | 6.69 | 0.59 | 11.28 | y | y |
|  | Fhod-B | 9.26 | 0.65 | 14.16 | y | y |
| Fhod-AΔ15 | Fhod-AΔ24 | 8.40 | 0.59 | 14.15 | y | y |
|  | Fhod-AΔ50 | 9.74 | 0.59 | 16.41 | y | y |
|  | Fhod-AΔ75 | 9.25 | 0.56 | 16.60 | y | y |
|  | Fhod-B | 11.82 | 0.62 | 19.02 | y | y |
| Fhod-AΔ24 | Fhod-AΔ50 | 1.34 | 0.59 | 2.26 | n | n |
|  | Fhod-AΔ75 | 0.85 | 0.56 | 1.52 | n | n |
|  | Fhod-B | 3.42 | 0.62 | 5.50 | y | y |
| Fhod-AΔ50 | Fhod-AΔ75 | 0.49 | 0.54 | 0.91 | n | n |
|  | Fhod-B | 2.08 | 0.61 | 3.41 | n | n |
| Fhod-AΔ75 | Fhod-B | 2.57 | 0.57 | 4.48 | y | n |

Statistical analysis for Figure 4.

Tables 5A-C present results of one-way ANOVA analysis with Tukey Kramer Post Hoc test.

Table 5A. Summary

| *Groups* | *Count* | *Sum* | *Average* | *Variance* |
| --- | --- | --- | --- | --- |
| Actin Alone | 30 | 252.04 | 8.40 | 6.36 |
| Fhod-A | 27 | 119.29 | 4.42 | 2.56 |
| Fhod-B | 29 | 144.04 | 4.97 | 2.79 |
| Fhod-E | 30 | 127.91 | 4.26 | 1.76 |

| Table 5B. ANOVA |  |  |  |  |  |  |
| --- | --- | --- | --- | --- | --- | --- |
| *Source of Variation* | *SS* | *df* | *MS* | *F* | *P-value* | *F crit* |
| Between Groups | 338.00 | 3 | 112.67 | 33.20 | 0.00 | 2.69 |
| Within Groups | 380.10 | 112 | 3.39 |  |  |  |
|  |  |  |  |  |  |  |
| Total | 718.10 | 115 |  |  |  |  |

Table 5C. Tukey Kramer Post Hoc Test

| *group 1* | *group 2* | *diff* | *SE* | *Q* | *p < 0.05*  *Qcrit (4,112)=3.69* | *p < 0.01*  *Qcrit (4,112)=4.50* |
| --- | --- | --- | --- | --- | --- | --- |
| Actin Alone | Fhod-A | 3.98 | 0.35 | 11.53 | y | y |
|  | Fhod-B | 3.43 | 0.34 | 10.12 | y | y |
|  | Fhod-E | 4.14 | 0.34 | 12.30 | y | y |
| Fhod-A | Fhod-B | 0.55 | 0.35 | 1.58 | n | n |
|  | Fhod-E | 0.15 | 0.35 | 0.45 | n | n |
| Fhod-B | Fhod-E | 0.70 | 0.34 | 2.07 | n | n |
